# Effectiveness and safety of immune checkpoint inhibitor monotherapy in advanced upper tract urothelial carcinoma: A multicenter, retrospective, real‐world study

**DOI:** 10.1002/cam4.5796

**Published:** 2023-03-23

**Authors:** Ruopeng Su, Zeyu Chen, Daoping Hong, Shuai Jiang, Yichu Yuan, Xingyun Cai, Hailong Hu, Changde Fu, Zhiyang Huang, Zhenyu Wang, Bing Zheng, Jian Huang, Zaoyu Wang, Yige Bao, Ming Cai, Jianming Guo, Minfeng Chen, Qiang Wei, Jiwei Huang, Wei Xue

**Affiliations:** ^1^ Department of Urology, Renji Hospital, School of Medicine Shanghai Jiao Tong University Shanghai China; ^2^ Department of Urology and Institute of Urology West China Hospital, Sichuan University Chengdu China; ^3^ Department of Urology, Xiangya Hospital Central South University Changsha China; ^4^ Department of Urology, Zhongshan Hospital Fudan University Shanghai China; ^5^ Department of Urology, The Second Affiliated Hospital, School of Medicine Zhejiang University Hangzhou China; ^6^ Department of Urology The Second Hospital of Tianjin Medical University Tianjin China; ^7^ Department of Urology Quanzhou First Hospital affiliated to Fujian Medical University Quanzhou China; ^8^ The Department of Urology The Second Affiliated Hospital of Nantong University Nantong China; ^9^ The Department of Urology, AnHui NO.2 Provincial People Hospital Hefei China; ^10^ Department of Pathology, Renji Hospital, School of Medicine Shanghai Jiao Tong University Shanghai China

**Keywords:** advanced UTUC, cisplatin‐ineligible, immune checkpoint inhibitor

## Abstract

**Introduction:**

The effectiveness and safety of immune checkpoint inhibitor (ICI) monotherapy in advanced upper tract urothelial carcinoma (UTUC) is less reported.

**Methods:**

In total, 106 consecutive advanced UTUC patients receiving ICI monotherapy were collected from nine high volume centers. Clinical outcomes were analyzed according to multiple parameters (e.g., treatment line, metastatic sites). Objective response rate (ORR), overall survival (OS) and progression‐free survival (PFS) were captured after ICI initiation.

**Results:**

With a median follow‐up of 12.0 months, 25 patients in the first‐line group and 15 patients in the second‐line group died of UTUC. We reported a median OS of 18.0 months, a median PFS of 5.0 months, and an ORR of 38.6% for patients in the first‐line group; a median OS of 10.0 months, a median OS of 4.0 months, and an ORR of 27.8% for patients in the second‐line group. Complete response was observed in two patients in the first‐line group and one patient in the second‐line group with a total complete response rate of 2.8%. In the univariate and multivariate analysis, visceral metastasis with a hazard ratio of 2.4 was associate with poor OS. The most common treatment‐related adverse events included fatigue (11.3%), pruritus (10.4%), and diarrhea (6.6%).

**Conclusions:**

This real‐world study suggests that ICI monotherapy is active and has acceptable toxic effects for unresectable or metastatic UTUC as first‐line therapy in cisplatin‐ineligible patients or second‐line therapy in platinum‐refractory patients.

## Graphical abstract

PD‐1 inhibitor is active and has acceptable toxic effects as a first‐line or second‐line treatment in unresectable or metastatic UTUC. PD‐1 inhibitors could be an alternative choice for UTUC patients who are ineligible for cisplatin..

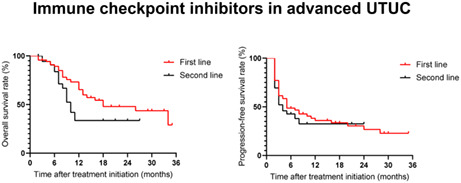

## 1 INTRODUCTION

Upper tract urothelial carcinoma (UTUC), which originates from the upper tract, pyelocaliceal cavities and ureters, accounts for 5%–10% of urothelial carcinoma (UC) cases worldwide and 20%–30% of UC cases in China.^1^, ^2^ The 5‐year disease‐specific survival of UTUC is between 61% and 76%, but stands at 30% for patients with high‐risk tumors (>pT3 and/or N+M+).^3^, ^4^, ^5^

The efficacy of platinum‐based chemotherapy is extrapolated from data collected in metastatic UC.^1^ An analysis of three randomized controlled trials suggested potential clinical benefit of these regimens in UTUC.^6^ There is a shift in the contemporary treatment paradigm for UTUC due to the breakthrough of immune checkpoint inhibitors (ICIs) in the treatment of UC.^7^, ^8^, ^9^, ^10^ Given that metastatic UTUC is characterized by impaired renal function, ICIs offer great promise for these patients.^11^ Several monoclonal antibodies including pembrolizumab, atezolizumab, avelumab, and nivolumab have been approved by the USA Food and Drug Administration (FDA) in the first‐ and second‐line settings of locally advanced UC and metastatic UC. However, data on ICIs clinical activity in advanced UTUC are limited to an analysis of registered clinical trials, which included only a minority of UTUC patients, ranging from 14% to 27%.^12^ This recent meta‐analysis showed that in the second‐line setting, the pooled objective response rate (ORR) from single‐arm studies was 21.2% (95% CI 12.5%–33.7%); a pooled analysis of two randomized controlled trials revealed no statistically significant difference in overall survival (OS) between patients treated with standard second‐line chemotherapy and those with ICIs despite a 24% reduction in the risk of death in patients treated with ICI.^12^ Two trials IMvigor210 (cohort 1) and KEYNOTE‐052 in the cisplatin‐ineligible first‐line setting reported ORR of 39% and 22% in the UTUC subgroup, respectively.^8^, ^9^ However, no progression‐free survival (PFS) or OS was reported.

Recently, tislelizumab and toripalimab were conditionally approved in the second‐line setting by China National Medical Products Administration (NMPA). Single agent tislelizumab or toripalimab is generally well tolerated and has promising antitumor activities in patients who have received prior platinum‐based chemotherapy.^13^, ^14^ In the second‐line setting, tislelizumab resulted in a median PFS of 2.1 months and a median OS of 9.8 months, while toripalimab resulted in a median PFS of 2.3 months and a median OS of 14.4 months.^13^, ^14^ Although the results were encouraging, UTUC patients accounted for less than half of the participants in these trials, and clinical outcomes were still lacking for this subgroup.

Geographical factors were involved in various UTUC morbidities worldwide. A high proportion of UTUC patients were enrolled in clinical trials that were conducted in China. Given the inherent heterogeneity of UTUC, it is important to further explore the efficacy in the real‐world setting. Here, we focused on understanding the efficacy and safety of ICIs in UTUC patients.

## 2 PATIENTS AND METHODS

### 2.1 Patients

After the study protocol was approved by the independent institutional review board, we retrieved data from the electronic medical records of UTUC patients who visited nine participating centers and received first‐ or second‐line anti‐PD‐1 antibody monotherapy between 2018 and 2021. We included adult patients aged 18 or older who had histologically or cytologically confirmed unresectable or metastatic renal pelvis and ureter urothelial cancer (including pure urothelial and mixed urothelial cell histology). The eligibility criteria for first‐line patients included ineligibility for cisplatin‐based therapy^1^ (defined as meeting at least one of the following criteria: Eastern Cooperative Oncology Group Performance Status 2 or 3, creatinine clearance <60 mL/min, grade ≥2 audiometric hearing loss, grade ≥2 peripheral neuropathy, or New York Heart Association Class III heart failure); second‐line was identified as follows: prior receipt of systemic chemotherapy for advanced disease (perioperative, platinum‐based chemotherapy with disease recurrence ≤12 months since completion was allowed); measurable lesion per Response Evaluation Criteria in Solid Tumors (RECIST, version 1.1)^15^; at least one imaging study of the target lesion after treatment. Exclusion criteria were treatment beyond second‐line, combination or sequential therapy; no imaging study was conducted after ICI initiation.

### 2.2 Treatment and procedures

ICIs used in this study were as follows: tislelizumab (*n* = 42, 40%), toripalimab (*n* = 31, 29%), pembrolizumab (*n* = 19, 18%), nivolumab (*n* = 5, 5%), sintilimab (*n* = 4, 4%), and camrelizumab (*n* = 5, 5%). Patients were treated with anti‐PD‐1 antibody by intravenous infusion once every 3 weeks with tislelizumab 200 mg, toripalimab 240 mg, pembrolizumab 200 mg, sintilimab 200 mg, or camrelizumab 200 mg and nivolumab 240 mg once every 2 weeks. Dose discontinuation was performed by the manufacturer's instructions to manage adverse events (AEs). Treatment continued until disease progressed, intolerable toxicities, or death.

### 2.3 Assessments

PD‐L1 expression was assessed in formalin‐fixed tumor samples at individual centers and re‐reviewed by a pathologist. Radiological evaluation was performed by computed tomography and/or magnetic resonance imaging of the abdomen, chest and brain as well as bone scintigraphy prior to initiation of treatment, and thereafter every 2–3 months by RECIST version 1.1, including complete response (CR), partial response (PR), stable disease (SD) and progressive disease (PD). ORR and disease control rate (DCR) were defined as CR+PR and CR+PR+SD, respectively. OS was defined as the duration from the onset of ICI therapy to death from any cause. PFS was defined as the duration from the onset of ICI therapy to disease progression or death. Duration of confirmed response (DOR), defined as the time from the first documented complete or PR to disease progression or death.

All observed AEs and the severity of AEs were evaluated using the National Cancer Institute Common Terminology Criteria for Adverse Events version 4.0.

### 2.4 Statistical analysis

Statistical analysis was performed using SPSS version 26.0 and GraphPad Prism 8. The PFS and OS curves were plotted using Kaplan–Meier method and survival outcomes were compared across groups using the log‐rank test. The Pearson chi‐squared test or Fisher exact test was used to compare categorical variables. The Cox proportional hazard model was used to evaluate the predictors of PFS or OS. *p* < 0.05 was considered significant.

## 3 RESULTS

### 3.1 Patient population

Between 2018 and 2021, 188 consecutive UTUC patients were identified from nine sites around China. After excluding ineligible patients, 106 patients were included in this analysis (Figure S1). Table 1 summarizes the demographic and baseline clinicopathological characteristics of the study patients. Of these patients, 70 cisplatin‐ineligible patients (66.0%) received ICI monotherapy as first‐line treatment. The remaining 36 patients were identified as platinum pretreated and received ICI alone in the second‐line setting.

**TABLE 1:** Baseline clinical characteristics and prior treatment of upper tract urothelial carcinoma patients.

Characteristic	All patients (*N* = 106)
Age, years (median, range)	67.5 (40–91)
Age ≥80 years	15 (14.2%)
Male sex	63 (59.4%)
ECOG	
0–1	60 (56.6%)
≥2	46 (43.4%)
Prior nephroureterectomy	72 (67.9%)
Metastatic disease	
Visceral sites	59 (55.7%)
Liver	23 (21.7%)
Bone	29 (27.4%)
Non‐visceral	47 (44.3%)
Primary tumor site	
Renal pelvis	54 (50.9%)
Ureter	48 (45.3%)
Both	4 (3.8%)
Treated as first line	70 (66.0%)
Treated as second line after chemotherapy	36 (34.0%)
Type of ICIs	
Tislelizumab	42 (39.6%)
Toripalimab	31 (29.2%)
Other agents	33 (31.1%)
PD‐L1 expression	
<1%	27 (25.5%)
≥1%	18 (17.0%)
Unknown	61 (57.5%)
eGFR	
≥60	39 (36.8%)
<60	67 (63.2.%)
Histology	
Pure urothelial	88 (83.0%)
Mixed urothelial	18 (17.0%)
Bajorin risk groups	
0	25 (23.6%)
1	56 (52.8%)
2	25 (23.6%)

Abbreviations: ECOG, Eastern Cooperative Oncology Group; ICI, immune checkpoint inhibitors; PD‐L1, programmed‐death ligand 1; UTUC, upper tract urothelial carcinoma.

### 3.2 Objective response rate

As shown in Table 2, confirmed ORR was 34.9% (37/106) of the total population. DCR was 58.5%. Despite limited enrollment, confirmed ORR (38.6%, 27/70) and DCR (61.4%, 43/70) in first‐line cisplatin‐ineligible patients were numerically higher than those in second‐line patients (ORR 27.8%, 10/36 and DCR 52.8%, 19/36). CR was observed in two patients in the first‐line group and one patient in the second‐line group with a total CR rate of 2.8%. The duration of response reached 28 months (95% CI NR–NR).

**TABLE 2:** Objective response and duration of response in all treated patients.

Subgroup	First line (*n* = 70)	Second line (*n* = 36)	Total (*n* = 106)
Confirmed objective response	27/70 (38.6%)	10/36 (27.8%)	37 (34.9%)
Complete response	2/70 (2.9%)	1/36 (2.8%)	3 (2.8%)
Partial response	25/70 (35.7%)	9/36 (25.0%)	34 (32.1%)
Stable disease	16/70 (22.9%)	9/36 (25.0%)	25 (23.6%)
Progressive disease	27/70 (38.6%)	17/36 (47.2%)	44 (41.5%)
Duration of response, months	–	–	28 (NR‐NR)
Age ≥80 years	–	–	5/15 (33.3%)
eGFR			
≥60	–	–	14/39 (35.9%)
<60	–	–	23/67 (34.3%)
Metastatic disease			
Visceral sites	–	–	17/59 (28.8%)
Liver	–	–	7/23 (30.4%)
Bone	–	–	7/29 (24.1%)
Non‐visceral	–	–	20/47 (42.6%)
PD‐L1 expression			
<1%	–	–	7/27 (25.9%)
≥1%	–	–	8/18 (44.4%)
Unknown	–	–	22/61 (36.1%)
Histology			
Pure urothelial	–	–	30/88 (34.1%)
Mixed urothelial	–	–	7/18 (38.9%)
Bajorin risk groups			
0	–	–	11/25 (44.0%)
1	–	–	19/56 (33.9%)
2	–	–	7/25 (28.0%)

Subgroup analysis by age, metastasis, PD‐L1 expression, histology, and Bajorin risk showed similar ORR in patients ≥80 years of age and mixed urothelial histology (Table 2). In line with previous reports, ORR was higher in patients without visceral metastasis and PD‐L1 expression >1%, but lower in patients with visceral metastasis and PD‐L1 expression <1%.^16^ In addition, the ORR was negatively associated with Bajorin risk score.

### 3.3 Survival outcomes

**FIGURE 1:**
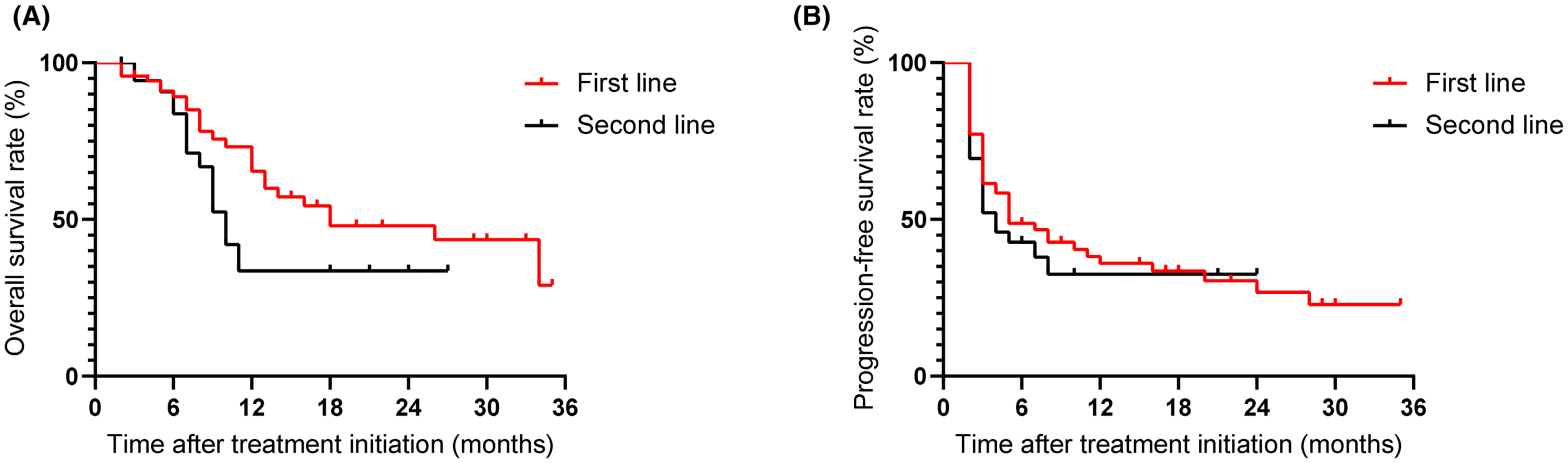
Overall survival (A) and progression‐free survival (B) of patients with advanced upper tract urothelial carcinoma stratified by treatment line of immune checkpoint inhibitors.

With a median follow‐up of 12 months, 25 patients in the first‐line group and 15 patients in the second‐line group died of UTUC. For cisplatin‐ineligible first‐line patients, the median OS was 18.0 months (95% CI 4.1–31.9), the 1‐year OS rate was 65.4%, and the median PFS was 5.0 months (95% CI 2.0–8.0; Figure 1A,B). For second‐line patients, the median OS was 10.0 months (95% CI 8.3–11.7), the 1‐year OS rate was 33.6%, and the median PFS was 4.0 months (95% CI 2.1–5.9; Figure 1A,B).

**FIGURE 2:**
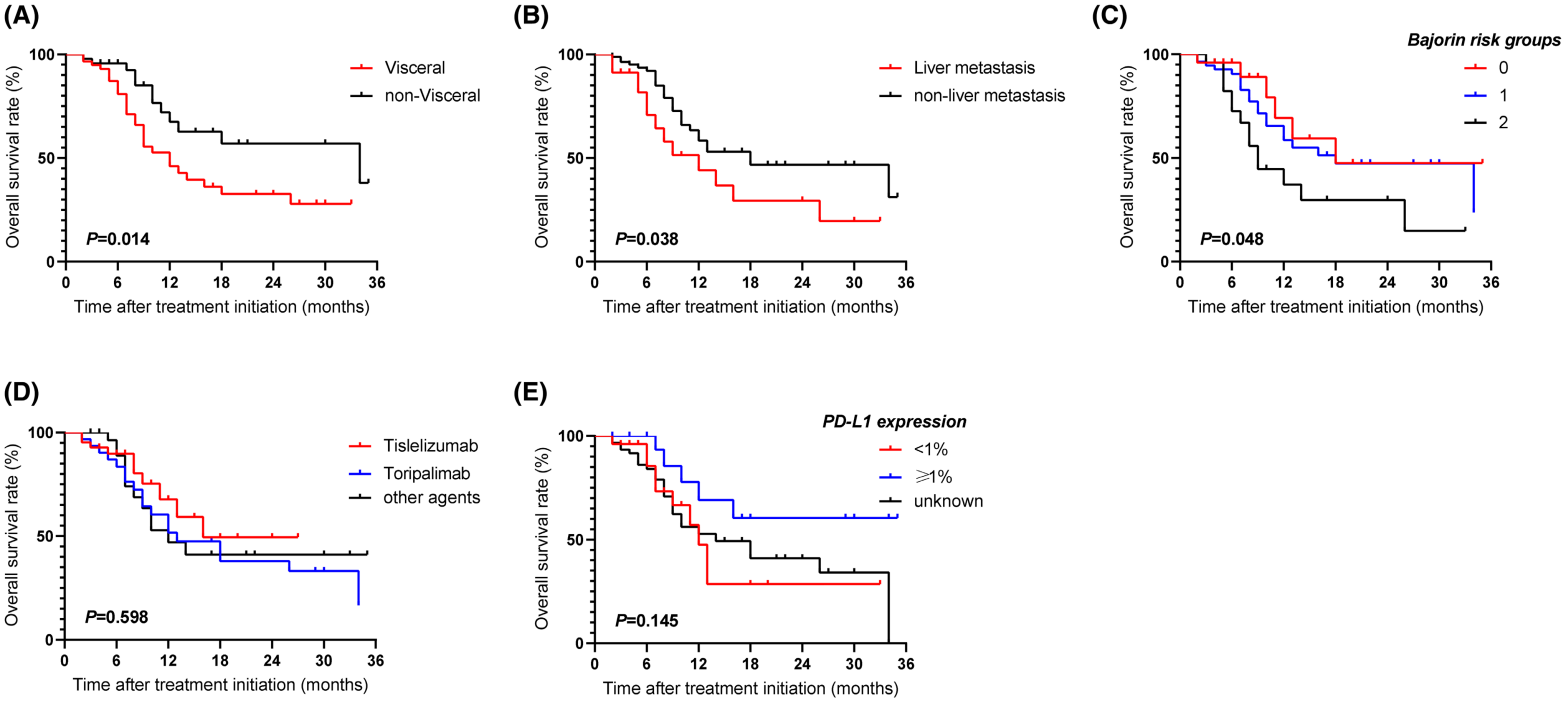
Subgroup analysis of upper tract urothelial carcinoma patients treated with immune checkpoint inhibitors (ICI) according to metastatic sites (A), the presence of liver metastasis (B), Bajorin risk groups (C), ICI agents (D), and PD‐L1 expression (E).

As shown in Table 3, the predictive factors of OS were analyzed by univariate and multivariate analysis. The OS in each subgroup was generally consistent with the overall population. Visceral metastasis, liver metastasis, and Bajorin risk were associated with significantly worse OS than those without (Figure 2A–C). Besides, OS with ICIs from different companies and PD‐L1 expression was comparable to the current sample size (Figure 2D,E).

**TABLE 3:** Univariate and multivariate analysis of associations of various parameters with overall survival.

Variable	Univariate analysis		Multivariate analysis	
	HR (95% CI)	*p*‐value	HR (95% CI)	*p*‐value
Age (>65 vs. ≤65 years)	0.828 (0.439–1.563)	0.560		
Gender (male vs. female)	0.857 (0.455–1.615)	0.633		
ECOG (≥2 vs.<2)	1.288 (0.692–2.399)	0.425		
eGFR (<60 vs. ≥ 60)	0.923 (0.486–1.753)	0.807		
Prior nephroureterectomy (yes vs. no)	0.750 (0.392–1.433)	0.383		
Primary tumor site				
Renal pelvis	1	0.399		
Ureter	1.544 (0.808–2.950)	0.189		
Both	1.613 (0.372–7.003)	0.523		
Metastatic sites (Visceral vs. non‐visceral)	2.300 (1.144–4.623)	0.019	2.300 (1.144–4.623)	0.019
Liver metastasis (yes vs. no)	1.973 (1.013–3.843)	0.046		
Histological subtype (Mixed urothelial vs. Pure urothelial)	1.027 (0.453–2.328)	0.949		
Type of PD‐1 antibody agent				
Tislelizumab	1	0.614		
Toripalimab	1.485 (0.678–3.254)	0.323		
Other agents	1.295 (0.558–3.006)	0.548		
Bajorin risk groups				
0	1	0.063		
1	1.326 (0.531–3.307)	0.546		
2	2.661 (1.017–6.964)	0.046		

### 3.4 Safety prolife

As shown in Table 4, the most common AEs included fatigue (11.3%), pruritus (10.4%), diarrhea (6.6%), decreased appetite (7.5%), rash (10.4%), hypothyroidism (7.5%), and increased ALT (6.6%) (Table 4). Grade 3–4 AEs included diarrhea (1.9%), rash (1.9%), increased ALT (1.9%) and AST (1.9%), and myocarditis (0.9%). AEs resulted in five ICI discontinuations.

**TABLE 4:** Treatment‐related adverse events.

Adverse event	Any grade	Grades 3 and 4
Any event	64 (60.4%)	11 (10.4%)
Event leading to discontinuation of treatment	5 (4.7%)	4 (3.8%)
Fatigue	12 (11.3%)	1 (0.9%)
Pruritus	11 (10.4%)	0 (0.0%)
Diarrhea	7 (6.6%)	2 (1.9%)
Decreased appetite	8 (7.5%)	0 (0.0%)
Rash	11 (10.4%)	2 (1.9%)
Hypothyroidism	8 (7.5%)	0 (0.0%)
Hyperthyroidism	3 (2.8%)	0 (0.0%)
Nausea	5 (4.7%)	0 (0.0%)
Pyrexia	6 (5.7%)	0 (0.0%)
ALT increased	7 (6.6%)	2 (1.9%)
AST increased	4 (3.8%)	2 (1.9%)
Constipation	4 (3.8%)	0 (0.0%)
Anemia	7 (6.6%)	2 (1.9%)
Interstitial pneumonia	4 (3.8%)	1 (0.9%)
Leucopenia	6 (5.7%)	0 (0.0%)
Neutrophil count decreased	5 (4.7%)	0 (0.0%)
lipase increase	7 (6.7%)	0 (0.0%)
Thrombocytopenia	3 (2.8%)	0 (0.0%)
Hypercholesteremia	3 (2.8%)	0 (0.0%)
Myocarditis	2 (1.9%)	1 (0.9%)
Bilirubin increase	1 (0.9%)	0 (0.0%)
Hyponatremia	1 (0.9%)	0 (0.0%)
Adrenal insufficiency	1 (0.9%)	0 (0.0%)
Arthralgia	1 (0.9%)	0 (0.0%)

## 4 DISCUSSION

To date, this study is the first real‐world retrospective study designed to explore the efficacy and safety of immunotherapy in advanced UTUC in China. Here, we presented ORR, PFS and OS to UTUC patients receiving ICI monotherapy. Promising efficacy and a similar safety profile were observed independently of the treatment line, although ICI monotherapy appeared to be more effective in the first‐line than in the second‐line. Univariate and multivariate analysis of OS suggested that visceral metastases, a significant adverse predictor of OS, indicated the need for more effective treatment. Since prospective clinical trials designed for UC generally enroll a minority of UTUC, the data provide meaningful insight into the specific efficacy and safety of ICI monotherapy in UTUC.

The European Association of Urology (EAU) guidelines recommended two ICIs in first‐line UTUC patients unfit for cisplatin depending on PD‐L1 status.^17^ The KEYNOTE‐052 trial enrolled 69 UTUC patients, accounting for 19% of the total population. The ORR was 22% for UTUC and 28% for lower tract UC.^9^ In the IMvigor‐210 trial cohort 1, a numerically higher ORR 39% was observed in 33 UTUC patients than ORR 16% for lower tract UC.^8^ No other data on UTUC was reported in these two trials. Three ICIs (pembrolizumab, avelumab and nivolumab) were recommended for the second‐line. Between 10% and 20% of UTUC patients were included in KETNOTE‐045, Javelin UC, and CheckMate‐275 studies.^10^, ^16^, ^18^ A meta‐analysis showed an ORR of 21.2% in pooled analysis.^12^ In the present study, a total of 70 first‐line and 36 second‐line patients were collected with ORR of 38.6% and ORR 27.8%, respectively. Interestingly, treated UTUC with ICI in the first‐line showed a higher ORR than in the second‐line.

The PFS of UTUC patients who received ICI monotherapy was rarely reported in clinical trials and retrospective studies. Our observations indicated that PFS after ICI initiation in first‐ and second‐line was 5.0 and 4.0 months, respectively. This is slightly higher than (2–3 months) in clinical trials enrolling first‐line cisplatin‐ineligible UC patients.^8^, ^9^ The median OS was 18.0 months in first‐line, which is comparable to (16.3 months) in IMvigor210 C1 and (11.3 months) KEYNOTE‐052. The median OS was 10.0 months in second‐line, which is also consistent with previous reports of UC.^10^, ^16^, ^19^ Overall, the PFS and OS data of the UTUC subset were limited in the pivotal studies, except for a median OS of 7.9 months in IMvigor 210 cohort 2 and 10.9 months in IMvigor 211.^20^ Esagian et al. retrospectively collected 746 UC patients involving a UTUC subset that received ICI monotherapy. UTUC patients were treated in the first‐line setting: ORR was 35% (95% CI 24%–48%), median PFS was 4.6 months (95% CI 2.5–8.3), and median OS was 13.4 months (95% CI 8.3–19.9); in the second‐line setting, ORR was 15% (95% CI 8%–26%), median PFS was 4.1 months (95% CI 2.8–5.9), and median OS 8.4 months (95% CI 5.3–14.0).^21^ Overall, ICI monotherapy showed comparable efficacy between our and previous studies.^21^

Since advanced UTUC patients usually present with renal impairment, they do not tolerate cisplatin combination therapy, which has promising efficacy in UC.^22^, ^23^ In addition, there is no specific prospective trial on the efficacy of platinum‐based chemotherapy in UTUC and chemotherapy is recommended based on extrapolated data from metastatic UC.^24^ Retrospective comparison between UTUC and lower tract UC indicated that the location of the primary tumor had little impact on the response rate and had comparable efficacy.^6^, ^24^ A Phase III study compared the efficacy of pembrolizumab and the investigator's choice of chemotherapy in 542 UC patients. OS was significantly longer in the pembrolizumab group than in the chemotherapy group with HR 0.73 (95% CI 0.59–0.91, *p* = 0.02). For UTUC patients, the exploratory subset analysis of this trial demonstrated prolonged OS in the pembrolizumab group (HR 0.53, 95% CI 0.28–1.01).^10^ This study shifted the paradigm of contemporary treatment strategy of UC in the post platinum‐based chemotherapy setting. ICI monotherapy showed two notable advantages in this group of patients: durable efficacy and a good safety profile. It was previously believed that advanced UC patients, especially those who had progressed from platinum‐based chemotherapy, had poor long‐term OS.^25^ Patients rarely survived more than 24 months.^26^ Administration of ICI increased ORR and provided meaningful OS benefits. The breakthrough of ICI is that one in five patients responding to ICI therapy shows prolonged DOR.^27^ In this study, we found that the 24‐month PFS rate was about 30% in the first‐ and second‐line, and the DOR was over 28 months.

In the present study, we retrospectively collected AEs in electronic medical record systems. The most common treatment‐related AEs included fatigue (11.3%), pruritus (10.4%), and diarrhea (6.6%). Serious AEs causing ICI discontinuation were infrequent. However, due to the inherent disadvantage of retrospective analysis, treatment‐related AEs might be underestimated.

UTUC has been identified as a highly heterogeneous disease.^17^ Genomic profile is inconsistent between UTUC and lower tract UC.^28^ PD‐L1 is recognized as a classic predictive factor of response to ICI. However, its role in UC is contradictory due to limited efficacy and several conflicting results.^8^, ^19^, ^29^ In our study, PD‐L1 status was available in 42.5% of patients, including 27 (25.5%) PD‐L1 < 1% and 18 (17.0%) PD‐L1 ≥ 1% patients. DACO 22C3 was used to identify PD‐L1 status. As shown in Table 2, a numerically higher ORR was observed in patients with PD‐L1 ≥ 1% than PD‐L1 < 1% (PD‐L1 ≥ 1% vs. PD‐L1 < 1%: 44.4% [8/18] vs 7/27 [25.9%]). Many issues remain to be explored in UTUC, such as the range of PD‐L1 ≥ 1% prevalence, consistency of different detection platforms, and the relationship between PD‐L1 status and prognosis. Lynch syndrome is prevalent in UTUC patients with a 4% positive rate, but is rarely found in lower tract UC.^30^ Multiple studies proved that patients with tumors harbor germline mutations in mismatch repair (MMR) genes might respond to ICI treatment. Screening for such mutations is meaningful for deciding on further treatment strategies in UTUC patients.^17^ Fibroblast growth factor receptor 3 (*FGFR3*) mutations are also more frequent in UTUC than lower tract UC.^31^ Such mutations are associated with better survival and lower grade tumors. As the rising of FGFR inhibitors in mUC, combination of an FGFR3 inhibitor with an ICI may be a promising strategy. Despite debate about whether UTUC and bladder UC are two distinct diseases, results from our and previous studies suggested similar efficacy after ICI monotherapy.^12^

Recently, many novel drugs, including erdafitinib, enfortumab vedotin, and sacituzumab govitecan, have emerged. Given the heterogeneity of UTUC, a combination strategy is promising in overcoming potential ICI resistance. Enfortumab vedotin plus pembrolizumab in first‐line cisplatin‐ineligible patients showed promising ORR PFS OS.^32^ The combination strategy consists of ICI and novel drugs that may improve the prognosis of UTUC patients, especially those with visceral metastases. Prospective Phase III clinical trials (NCT04223856 and NCT05092958) are underway.

We previously compared the efficacy and safety of PD‐1 inhibitors and carboplatin combined with gemcitabine in the first‐line treatment of cisplatin‐unfit UTUC patients.^33^ Our results support the role of PD‐1 inhibitors with comparable survival outcomes, longer DOR, and lower toxicity than carboplatin‐gemcitabine for cisplatin‐ineligible patients, although PD‐1 inhibitors may not surpass cisplatin dominance in the treatment of those eligible patients. In this study, we further explore the effect of ICIs in a second‐line setting and provide a full spectrum of efficacy and safety of ICI monotherapy for unresectable or metastatic UTUC as a first‐line therapy in cisplatin‐ineligible patients or second‐line therapy in platinum‐refractory patients. Due to the rarity of advanced UTUC, our studies have to integrate the results of patients receiving different ICIs. Future studies may focus on one specific agent, particularly a broadly applicable one such as pembrolizumab or atezolizumab, and prospectively investigate its role in the treatment of a large sample cohort of advanced UTUC.

The major limitation of this study included relatively short follow‐up, retrospective design, and potential selection bias. Short follow‐up may affect the interpretation of survival outcomes including OS and PFS. While the ORR and AEs observed were much more convincing. In addition, most of the ICIs used in our study were tislelizumab, toripalimab, sintilimab, and camrelizumab that were only available in China, which limits the broad application of our results. Nevertheless, the strength of this study is its provision of a large cohort of real‐world patients from nine participating high‐volume medical centers.

## 5 CONCLUSION

In conclusion, ICI monotherapy is active and has acceptable toxic effects for unresectable or metastatic UTUC as first‐line therapy in cisplatin‐ineligible patients or second‐line therapy in platinum‐refractory patients.

## AUTHOR CONTRIBUTIONS

**Ruopeng Su:** Data curation (equal); formal analysis (equal); investigation (equal); methodology (equal); visualization (equal); writing – review and editing (equal). **Zeyu Chen:** Data curation (equal); formal analysis (equal); investigation (equal); visualization (equal). **Daoping Hong:** Data curation (equal); formal analysis (equal); investigation (equal); resources (equal). **Shuai Jiang:** Data curation (equal); formal analysis (equal); investigation (equal); resources (equal). **Yichu Yuan:** Data curation (equal); formal analysis (equal); investigation (equal). **Xingyun Cai:** Data curation (equal); formal analysis (equal); investigation (equal). **Hai‐Long Hu:** Data curation (equal); formal analysis (equal); investigation (equal); resources (equal); validation (equal). **Changde Fu:** Investigation (equal); validation (equal). **Zhiyang Huang:** Investigation (equal); validation (equal). **Zhenyu Wang:** Investigation (equal); validation (equal). **Bing Zheng:** Investigation (equal); validation (equal). **Jian Huang:** Investigation (equal); validation (equal). **Zaoyu Wang:** Investigation (equal); validation (equal). **Yige Bao:** Investigation (equal); resources (equal); supervision (equal); validation (equal). **Ming Cai:** Investigation (equal); supervision (equal). **Jianming Guo:** Investigation (equal); supervision (equal); validation (equal). **Minfeng Chen:** Investigation (equal); resources (equal); supervision (equal). **Qiang Wei:** Investigation (equal); resources (equal); supervision (equal). **Jiwei Huang:** Conceptualization (equal); data curation (equal); formal analysis (equal); funding acquisition (equal); supervision (equal); writing – original draft (equal). **Wei Xue:** Conceptualization (equal); investigation (equal); project administration (equal); resources (equal); supervision (equal).

## FUNDING INFORMATION

This study was supported by the Natural Science Foundation of Shanghai (grant number 21ZR1438900), the Incubating Program for Clinical Research Innovation of Renji Hospital (grant number PYIII20‐07), and Basic Oncology Research Program from Bethune Charitable Foundation (BCF‐NH‐ZL‐20201119‐024).

## CONFLICT OF INTEREST STATEMENT

The authors declare no potential conflicts of interest.

## PATIENT CONSENT FOR PUBLICATION

This retrospective, multicenter study has obtained informed consent from participating patients.

## ETHICS APPROVAL

This retrospective, multicenter, real‐world study was conducted in accordance with the ethical standards of the Declaration of Helsinki and approved by the independent ethics committee at each participating center (ethics approval ID:KY2021‐102).

## Supporting information


Figure S1:

## Data Availability

Further details that support the findings of this study are available from the corresponding authors upon request.
