# Supplementary figures and images for: Effectiveness and safety of immune checkpoint inhibitor monotherapy in advanced upper tract urothelial carcinoma: A multicenter, retrospective, real‐world study
Source: Cancer Med. 2023 Mar 23;12(9):10587–96. doi: 10.1002/cam4.5796 (PMC10225242; doi:10.1002/cam4.5796)

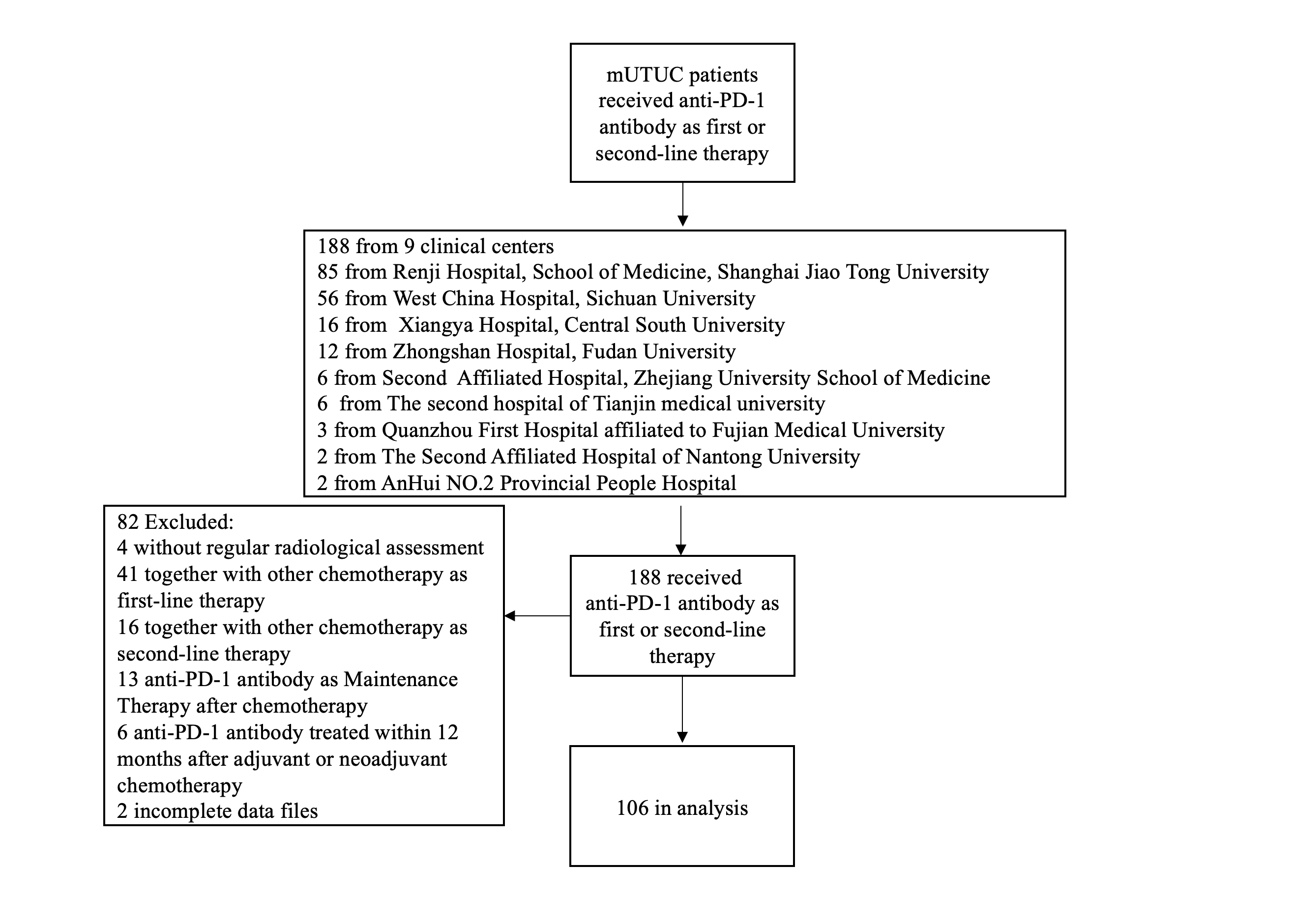

Supplement: Supplementary file 1 — Figure S1: [file CAM4-12-10587-s001.png]
